# Cholera Vaccine: Recommendations of the Advisory Committee on Immunization Practices, 2022

**DOI:** 10.15585/mmwr.rr7102a1

**Published:** 2022-09-30

**Authors:** Jennifer P. Collins, Edward T. Ryan, Karen K. Wong, Matthew F. Daley, Adam J. Ratner, Grace D. Appiah, Pablo J. Sanchez, Bruce J. Gutelius

**Affiliations:** ^1^Division of Foodborne, Waterborne, and Environmental Diseases, National Center for Emerging and Zoonotic Infectious Diseases, CDC; ^2^Harvard Medical School, Massachusetts General Hospital, Boston, Massachusetts; ^3^Institute for Health Research, Kaiser Permanente Colorado, Denver, Colorado; ^4^New York University Grossman School of Medicine, New York, New York; ^5^Division of Global Migration and Quarantine, National Center for Emerging and Zoonotic Infectious Diseases, CDC; ^6^Nationwide Children’s Hospital, The Ohio State University College of Medicine, Columbus, Ohio

## Abstract

This report summarizes all recommendations from CDC’s Advisory Committee on Immunization Practices (ACIP) for the use of lyophilized CVD 103-HgR vaccine (CVD 103-HgR) (Vaxchora, Emergent BioSolutions, Gaithersburg, MD) in the United States. The live attenuated oral cholera vaccine is derived from

Vibrio cholerae *O1 and is administered in a single dose. Cholera is a toxin-mediated bacterial gastrointestinal illness caused by toxigenic* V. cholerae *serogroup O1 or, uncommonly, O139. Up to 10% of infections manifest as severe cholera (i.e., cholera gravis), profuse watery diarrhea that can cause severe dehydration and death within hours. Fluid replacement therapy can reduce the fatality rate to <1%. Risk factors for cholera gravis include high dose exposure, blood group O, increased gastric pH (e.g., from antacid therapy), and partial gastrectomy. Cholera is rare in the United States, but cases occur among travelers to countries where cholera is endemic or epidemic and associated with unsafe water and inadequate sanitation. Travelers might be at increased risk for poor outcomes from cholera if they cannot readily access medical services or if they have a medical condition that would be worsened by dehydration, such as cardiovascular or kidney disease. This report describes previously published ACIP recommendations about use of CVD 103-HgR for adults aged 18–64 years and introduces a new recommendation for use in children and adolescents aged 2–17 years. ACIP recommends CVD 103-HgR, the only cholera vaccine licensed for use in the United States, for prevention of cholera among travelers aged 2–64 years to an area with active cholera transmission. Health care providers can use these guidelines to develop the pretravel consultation for persons traveling to areas with active cholera transmission.*

## Introduction

Cholera is an acute, watery diarrheal illness, primarily caused by toxigenic *V. cholerae* serogroup O1 that can be severe and rapidly fatal without proper treatment. CVD 103-HgR, a single-dose, live attenuated oral cholera vaccine derived from *V. cholerae* O1, is the only cholera vaccine licensed for use in the United States. In June 2016, the Food and Drug Administration (FDA) approved CVD 103-HgR for the prevention of cholera caused by *V. cholerae* O1 in adults aged 18–64 years traveling to cholera-affected areas (*1*). In June 2016, ACIP voted to recommend use of CVD 103-HgR for prevention of cholera among adult travelers to areas with active cholera transmission (*2*). In December 2020, FDA extended the approved usage to include children and adolescents aged 2–17 years (*3*). In February 2022, ACIP voted to recommend the use of CVD 103-HgR for children and adolescents aged 2–17 years traveling to an area with active cholera transmission. ACIP recommends CVD 103-HgR for prevention of cholera among travelers aged 2–64 years to an area with active cholera transmission. Health care providers can use these guidelines to develop the pretravel consultation for persons traveling to areas with active cholera transmission.

## Background Information on Cholera

Cholera is a toxin-mediated bacterial gastrointestinal illness caused by toxigenic *V. cholerae* serogroup O1 or, uncommonly, O139. *V. cholerae* O1 has an aquatic reservoir, and human infection occurs via ingestion of contaminated water or food (*4*,*5*). Direct fecal-oral transmission can occur, but secondary cases from person-to-person transmission are rare if sanitation is adequate (*4*). The incubation period usually ranges from hours to a few days (*4*,*5*).

Infection with toxigenic *V. cholerae* O1 can cause a range of symptoms. Up to 10% of infections manifest as cholera gravis, profuse watery diarrhea that can cause severe dehydration and death within hours without treatment (*4*). The case-fatality rate ranges from as high as 50% without treatment to <1% with appropriate fluid replacement therapy (*6*). Fluid replacement should be based on the degree of volume depletion and ongoing fluid losses. Oral rehydration solution can be used for milder cases, whereas intravenous fluids are needed for severe dehydration or hypovolemic shock. Antibiotics, which have been shown to shorten the duration of illness and fluid requirements, are an adjunct for severely ill patients (*4*).

A definitive diagnosis of cholera is based on culture of stool or rectal swab specimens. Special transport and culture media not routinely used for stool cultures are needed to isolate *V. cholerae*. Other stool-based diagnostic tests include antigen tests, darkfield microscopy, and molecular assays. Although certain multiplex gastrointestinal panels used in clinical settings have a target for *V. cholerae*, these tests are insufficient for diagnosis without serogroup information and confirmation of toxin production.

Recommended measures to prevent cholera infection in cholera-endemic areas include using safe water (i.e., for drinking, brushing teeth, washing and preparing food, and making ice) and eating safe food (i.e., those that are packaged or fully cooked and served hot). Other important prevention measures include washing hands often with soap and safe water and using sanitary methods to dispose of stool (https://www.cdc.gov/cholera/preventionsteps.html).

### Global Epidemiology

Cholera is endemic in approximately 50 countries and can cause large epidemics (*7*). Cholera epidemics are associated with unsafe water and inadequate sanitation. An estimated 1.3–4.0 million cases of cholera and 21,000–143,000 deaths occur worldwide each year (*5*). CDC’s Travelers’ Health Branch monitors areas with active cholera transmission and updates the list of affected countries monthly (https://wwwnc.cdc.gov/travel/news-announcements/cholera-vaccine-for-travelers).

### Epidemiology in the United States and Exposure Risk Among Travelers

Cholera is rare in the United States, although likely underreported. Most U.S. cases occur among travelers to countries where cholera is endemic or epidemic (*8*). Domestically acquired cases are usually associated with consuming undercooked seafood from coastal waters where *V. cholerae* species are endemic in the environment, such as along the Gulf Coast (*8*). During 2012–2018, a total of 64 cases of cholera in the United States were reported to CDC’s Cholera and Other Vibrio Illness Surveillance system; 56 (88%) were travel associated. Five cases (8%) occurred in children and adolescents aged 2–17 years; all were travel associated (*9*).

Cholera infections among U.S. international travelers are rare because most do not travel to areas with active cholera transmission and are able to access safe water and food at their destination (*10*). Persons at higher risk for exposure to toxigenic *V. cholerae* O1 include persons living in or traveling to cholera-affected areas for extended periods, travelers visiting friends and relatives, health care personnel, and cholera outbreak response workers (*8*,*11*–*13*). Cholera can be prevented by exclusive use of safe water and food, frequent handwashing, and adequate sanitation.

### Risk for Poor Outcomes from Cholera

Risk factors for cholera gravis include high-dose exposure, increased gastric pH (e.g., from antacids, proton-pump inhibitory therapy, or partial gastrectomy), and blood group O (*14*,*15*). Many travelers will not know their blood group at the time of consultation; however, an estimated 45% of persons in the United States have blood group O (*16*). Pregnancy is a risk factor for poor outcomes from cholera infection (*17*). Travelers also can be at increased risk for poor outcomes from cholera if they cannot readily access medical services or if they have a medical condition that would be worsened by dehydration (e.g., cardiovascular or kidney disease).

### Cholera Vaccine

CVD 103-HgR is a single-dose, live attenuated oral cholera vaccine derived from *V. cholerae* O1. In June 2016, FDA approved CVD 103-HgR for the prevention of cholera caused by *V. cholerae* O1 in adults aged 18–64 years traveling to cholera-affected areas (*1*). In December 2020, FDA extended the approved usage to include children and adolescents aged 2–17 years (*3*). CVD 103-HgR is the only cholera vaccine licensed for use in the United States; all ACIP recommendations in this report pertain to this formulation.

A previous formulation of CVD 103-HgR with identical phenotypic and genomic properties was licensed in other high-income countries under the trade names Orochol and Mutachol (*18*). Production was discontinued in 2003 for business reasons (i.e., not for safety or efficacy concerns) (*2*). Four other oral cholera vaccines (Dukoral, Shanchol, Euvichol, and Euvichol-Plus) are prequalified by the World Health Organization but are not available in the United States (*19*).

### Immunologic Response to Cholera and Cholera Vaccine

Protection against cholera is largely serogroup specific, and serogroup specificity is defined by the O-specific polysaccharide component of *V. cholerae* lipopolysaccharide (*20*). Infection with *V. cholerae* O1 protects against symptomatic reinfection, at least for a few years (*21*,*22*). A precise and easily measurable long-term correlate of protection against cholera remains elusive because protection is thought to be mediated by immune responses at the intestinal mucosal surface (*20*). The serum vibriocidal antibody response is commonly used as a correlate of protection, although it is most likely a surrogate marker (*23*). The vibriocidal response is primarily directed to the O-specific polysaccharide component of *V. cholerae* (*20*,*24*). O-specific polysaccharide-specific memory B-cells also are associated with long-term protection but are more challenging to measure than serum antibodies (*25*).

## Methods

Two ACIP work groups related to cholera vaccination met regularly to review relevant data and prepare draft policy recommendations for ACIP consideration. The Adult Cholera Vaccine Work Group (active August 2015–June 2016) focused on use of CVD 103-HgR among adults aged 18–64 years. The Pediatric Cholera Vaccine Work Group (active October 2020–February 2022) focused on use of CVD 103-HgR among children and adolescents aged 2–17 years. The work groups included two ACIP voting members, ex officio and liaison organization representatives, and consultants. The work groups had members with expertise in cholera, travel medicine, immunology, infectious diseases, epidemiology, obstetrics and gynecology (adult work group only), pediatrics (pediatric work group only), public health, and vaccination safety. The work groups convened via regularly scheduled teleconferences to discuss the epidemiology and immunology of cholera, data on the safety and efficacy of CVD 103-HgR in the target population, feasibility of administration, and other considerations.

ACIP used the Grading of Recommendations, Assessment, Development and Evaluation (GRADE) approach to evaluate the certainty of evidence for selected benefits and harms prespecified by each work group (Table 1) (*26*). For both age groups, ACIP considered evidence regarding safety and immunogenicity of the available formulation of CVD 103-HgR (*27*–*31*). For adults aged 18–64 years, ACIP also considered evidence regarding the efficacy of the current formulation of CVD 103-HgR (data were limited to adults aged 18–45 years) and evidence regarding efficacy and safety of the previously available formulation of CVD 103-HgR (*2*,*32*).

**TABLE 1:** Outcomes and rankings used for GRADE assessments of lyophilized CVD 103-HgR vaccine, by age group — Advisory Committee on Immunization Practices Cholera Vaccine Work Groups, United States, 2015–2022

Age group	Outcome	Ranking
**Adult (18–64 years)**	Cholera death	Critical
	Cholera diarrhea, life threatening	Critical
	Cholera diarrhea, severe	Critical
	Cholera diarrhea, any severity	Important
	Induction of vibriocidal antibody response	Important
	Serious adverse events	Critical
	Systemic adverse events	Critical
	Impact on effectiveness of coadministered vaccines and medications	Critical
**Pediatric (2–17 years)**	Cholera diarrhea, moderate to severe	Critical
	Cholera diarrhea, any severity	Critical
	Serious adverse events	Critical
	Nonserious adverse events	Important

**Abbreviation:** GRADE = Grading of Recommendations, Assessment, Development, and Evaluation.

In February 2018, ACIP adopted the Evidence to Recommendations (EtR) framework (https://www.cdc.gov/vaccines/acip/recs/grade/etr.html) to facilitate all deliberations related to its recommendations and provide transparency regarding factors that influence policy decisions (*33*). ACIP deliberations regarding use of CVD 103-HgR in adults aged 18–64 years preceded adoption of the EtR framework; therefore, recommendations for this age group did not follow the EtR framework. ACIP deliberations regarding use of CVD 103-HgR in children and adolescents aged 2–17 years followed adoption of the EtR framework and considered the importance of cholera as a public health problem, benefits and harms of CVD 103-HgR, values of the respective population, acceptability, feasibility, resource use, and equity. Expert judgment was considered when evidence was not available; adult and pediatric Cholera Vaccine Work Group presentations to ACIP were examined (Table 2).

**TABLE 2:** Timeline of Cholera Vaccine Work Group presentations to the Advisory Committee on Immunization Practices — United States, 2015–2022

Work group	ACIP meeting topic	Date of presentation
**Adult**	Overview of cholera epidemiology and lyophilized CVD 103-HgR vaccine	October 2015
	GRADE assessment	February 2016
	Proposed recommendations, public comment, and vote	June 2016
**Pediatric**	Overview of cholera epidemiology and lyophilized CVD 103-HgR vaccine	February 2021
	GRADE assessment and EtR framework	January 2022
	EtR summary, considerations for use, proposed policy option, public comment, and vote	February 2022

**Abbreviations:** ACIP = Advisory Committee on Immunization Practices; EtR = Evidence to Recommendations; GRADE = Grading of Recommendations, Assessment, Development, and Evaluation.

### Available Evidence

The efficacy of the current formulation of CVD 103-HgR against moderate or severe diarrhea (defined as cumulative fecal output >3 L after oral toxigenic *V. cholerae* O1 experimental challenge) in adults aged 18–45 years is estimated to be 90% at 10 days after vaccination and 80% at 3 months after vaccination (*32*). A randomized controlled trial of the previous formulation with identical phenotypic and genomic properties (manufacture production discontinued in 2003) demonstrated similar efficacy (*34*). No studies directly assessed vaccine efficacy among children and adolescents aged 2–17 years; only immunogenicity data were available. In both adults aged 18–64 years and children and adolescents aged 2–17 years, the current formulation of CVD 103-HgR induced serum vibriocidal antibody seroconversion (a fourfold or more rise in titer) in >93% of recipients on day 11 (*27*,*29*–*31*).

In clinical trials conducted using the current formulation, no vaccine-related serious adverse events were reported among participants aged 2–64 years (*27*,*29*–*31*). In clinical trials among adults aged 18–45 years, solicited adverse events were more commonly reported by CVD 103-HgR recipients than by placebo (i.e., buffer alone) recipients ≤7 days after vaccination and included tiredness (31.3% versus 27.4%; mostly mild or moderate), headache (28.9% versus 23.6%; mostly mild), nausea or vomiting (18.3% versus 15.2%; mostly mild), and diarrhea (3.9% versus 1.2%; mostly mild) (*31*,*35*). In the clinical trials among children and adolescents aged 2–17 years, any solicited adverse event ≤7 days after vaccination was reported by 55.1% of vaccine recipients and 50.7% of placebo recipients. Solicited adverse events more commonly reported by vaccine than placebo recipients aged 2–17 years included tiredness (35.7% versus 30.7%; mostly mild), headache (27.4% versus 25.3%; mostly mild), abdominal pain (27.8% versus 18.7%; mostly mild), and lack of appetite (21.4% versus 14.7%; mostly mild) (*27*,*29*,*35*).

### GRADE Quality Assessment (Certainty) of the Evidence

#### Children and Adolescents Aged 2–17 years

For prevention of cholera diarrhea (moderate to severe; any severity), the available body of evidence consisted of randomized controlled trials of the current formulation of CVD 103-HgR; this evidence indirectly assessed vaccine effectiveness via immunobridging and was deemed GRADE evidence type 2 (i.e., moderate certainty). For safety outcomes, the data were more limited; the small sample size (468 vaccine recipients and 75 placebo recipients) resulted in imprecise pooled effect estimates, and the final evidence was type 3 (i.e., low certainty) (https://www.cdc.gov/vaccines/acip/recs/grade/cholera-CVD-103-HgR-child-2-17-years.html).

#### Adults Aged 18–64 Years

Through the systematic review and quality assessment, the adult work group in 2016 found high-certainty evidence that the vaccine is highly effective and low-certainty evidence that it is safe. The body of evidence, which included studies with the currently available CVD 103-HgR formulation and studies with oral toxigenic *V. cholerae* O1 challenge, indicated high vaccine efficacy and was judged to be GRADE evidence type 1 (i.e., high certainty). For safety outcomes, the data were more limited because relatively few persons had received the current vaccine formulation. Few studies evaluated coadministration with other vaccines or medications; all used the previous formulation of CVD 103-HgR (*36*). Because of these limitations, the GRADE evidence rating for safety outcomes was judged to be type 3 (i.e., low certainty) (https://www.cdc.gov/vaccines/acip/recs/grade/cholera-CVD-103-HgR.html).

## Rationale for Cholera Vaccine Recommendations

Both ACIP work groups assessed the risk for cholera in U.S. travelers through review of the epidemiology of cholera and consideration of expert judgment. Cholera is rare among travelers returning to the United States from cholera-affected areas, and cholera is treatable if medical services are readily accessible (*6*,*8*). However, certain populations are at higher risk for severe toxigenic *V. cholerae* O1 infection and adverse outcomes (*14*,*15*,*17*). A traveler’s risk for acquiring cholera and developing severe illness might not be clear at the pretravel visit with their health care provider. Although cholera is rare, both work groups concluded that a safe and effective vaccine that can prevent a potentially severe cholera infection can benefit certain travelers. Additional information can be found in the EtR framework regarding children and adolescents aged 2–17 years (https://www.cdc.gov/vaccines/acip/recs/grade/cholera-CVD-103-HgR-child-2-17-years-etr.html) and in the previously published ACIP recommendations for adults aged 18–64 years (*2*).

## Recommendations for Prevention of Severe Cholera Among Travelers

### Personal Protective Measures

All travelers to cholera-affected areas should consume safe food and water, wash their hands often with soap and safe water, and follow recommended sanitation practices (https://www.cdc.gov/cholera/preventionsteps.html). Travelers who develop severe diarrhea should seek prompt medical care, particularly fluid replacement.

### Population Recommended for Vaccination with CVD 103-HgR

ACIP recommends CVD 103-HgR for travelers aged 2–64 years to an area of active cholera transmission. An area of active cholera transmission is defined as a province, state, or other administrative subdivision within a country with endemic or epidemic cholera caused by toxigenic *V. cholerae* O1 and includes areas with cholera activity within the past year that are prone to recurrence of cholera epidemics (*2*). An area of active cholera transmission does not include areas where only rare imported or sporadic cases have been reported. CDC’s Travelers’ Health Branch monitors areas with active cholera transmission and updates the list of affected countries monthly (https://wwwnc.cdc.gov/travel/news-announcements/cholera-vaccine-for-travelers).

CVD 103-HgR is not recommended for travelers who are not visiting areas with active cholera transmission. Most travelers from the United States do not visit areas with active cholera transmission (https://wwwnc.cdc.gov/travel/diseases/cholera#areas). No country requires vaccination against cholera as a condition for entry.

### CVD 103-HgR Booster Doses

No data exist about the safety or efficacy of preventing cholera with booster doses of the currently licensed CVD 103-HgR. The duration of protection conferred by the primary dose beyond the 3-month period evaluated in adults aged 18–45 years is unknown. ACIP does not have a recommendation regarding use of booster doses.

In an open label study of the previous formulation, 31 Swiss adults aged 23–48 years were given a single booster dose 15 or 24 months after the initial dose (*37*). One patient reported an adverse reaction (i.e., moderate diarrhea). Efficacy was not directly assessed, and a minority (i.e., approximately 25%) of participants experienced serum vibriocidal antibody seroconversion (a fourfold or more rise in titer) after the booster dose. The relation between serum vibriocidal antibody levels and efficacy has not been established beyond 3 months after vaccination.

### Administration of CVD 103-HgR

Health care providers should see the package insert for detailed administration instructions (*35*). CVD 103-HgR should be given ≥10 days before travel to an area with active cholera transmission. Recipients should avoid consuming food or drinks for 60 minutes before or after vaccine administration.

Administration instructions differ for recipients aged 2–5 years versus recipients aged 6–64 years. For all recipients, dose preparation begins with reconstituting the buffer sachet in 100 mL of cold or room temperature purified noncarbonated, nonflavored bottled or spring water. Tap water should not be used because it contains chlorine that can affect the viability of orally ingested live attenuated bacterial vaccines. For recipients aged 6–64 years, mix the active component (i.e., lyophilized *V. cholerae* CVD 103-HgR) with the full volume of reconstituted buffer solution (100 mL). For children aged 2–5 years, discard one half of the reconstituted buffer solution (50 mL) before adding the active component; this step reduces the vaccine volume (i.e., to aid consumption) while maintaining the specified potency (*38*).

To improve palatability, approximately all (93%) children and adolescents aged 2–17 years in the clinical trial added Pure Via brand stevia (1 g), a plant-derived sweetener, to the reconstituted CVD 103-HgR (*39*). During the January 12, 2022 ACIP meeting, the manufacturer presented unpublished data demonstrating that CVD 103-HgR should not be mixed with foods and drinks (e.g., rice cereal, applesauce, apple juice, or milk), because of excessive foaming, or with medicine flavorings that contain propylene glycol, which is bactericidal (*38*). The data support that the vaccine can be mixed with sucrose (table sugar, 1–4 g or ¼ to 1 teaspoon) or stevia sweetener (1 g or 1 packet; brands tested: Pure Via, Sweet Additions, Truvia, Splenda Naturals, and Sweetleaf) without the vaccine potency dropping below the minimum specification (*38*). Health care providers can consider administering CVD 103-HgR with sucrose or stevia sweetener to aid consumption of the full dose, although guidance regarding the use of sweeteners is not included in the package insert at the time of this publication.

### Coadministration of Other Medications or Vaccines with CVD 103-HgR

**Antibiotics.** Because the immune response to CVD 103-HgR relies on the live attenuated vaccine organisms replicating within the small intestine, antibiotics administered before or after the vaccine might diminish the vaccine’s effectiveness. The outcome will depend on factors such as the timing, half-life, and spectrum of the antibiotic. The optimal interval between CVD 103-HgR and receipt of antibiotics is unknown.

The package insert specifies that CVD 103-HgR should not be given to patients who have received oral or parenteral antibiotics during the preceding 14 days (*35*). A duration of fewer than 14 days between stopping antibiotics and giving CVD 103-HgR might be acceptable under certain circumstances, such as if travel cannot be avoided before 14 days have elapsed after stopping antibiotics.

The package insert does not specify an optimal minimum duration between completion of CVD 103-HgR and starting antibiotics (*35*). In certain circumstances, antibiotics might be clinically necessary after the vaccine (i.e., to treat an unrelated infection). Approximately all (>93%) vaccine recipients aged 2–45 years had vibriocidal antibody seroconversion ≤10 days after vaccination (*27*,*29*,*31*).

**Antimalarials.** Chloroquine might diminish the immune response to CVD 103-HgR (*35*). The manufacturer recommends that CVD 103-HgR be administered ≥10 days before starting chloroquine. Doxycycline, a tetracycline antibiotic, is often used for malaria prophylaxis. The optimal duration between completion of CVD 103-HgR and starting doxycycline is unknown.

**Other Vaccines.** No data are available on concomitant administration of the currently licensed CVD 103-HgR with other vaccines, including the enteric-coated oral live-attenuated typhoid vaccine (Ty21a, marketed as Vivotif [Emergent BioSolutions, Gaithersburg, MD]). The expert opinion of the ACIP work group postulated that CVD 103-HgR buffer might interfere with the enteric-coated Ty21a formulation and concluded that taking the first Ty21a dose ≥8 hours after ingestion of CVD 103-HgR might decrease potential interference of the vaccine buffer with the Ty21a vaccine.

### Contraindications, Precautions, and Other Considerations for Use of CVD 103-HgR

**Allergy.** CVD 103-HgR should not be administered to persons with a history of severe allergic reaction (e.g., anaphylaxis) to any component of this vaccine or to any cholera vaccine.

**Age.** No data exist about the safety and effectiveness of the currently available CVD 103-HgR vaccine in children aged <2 years or in adults aged ≥65 years.

**Pregnancy and Breastfeeding.** No data exist on use of the currently licensed CVD 103-HgR during pregnancy or while breastfeeding. Prospective travelers who are pregnant and their clinicians should consider the risks associated with traveling to areas with active cholera transmission. The *V. cholerae* O1 vaccine strain is not absorbed systemically; thus, maternal exposure to the vaccine is not expected to result in exposure to the fetus or breastfed infant to the vaccine. However, the vaccine strain might be shed in stool for ≥7 days after vaccination, and theoretically, the vaccine strain could be transmitted to an infant during vaginal delivery. A breastfed infant theoretically could receive benefit from maternally derived vaccine antibodies present in maternal milk.

**Persons with Altered Immunocompetence.** No data exist on use of the currently licensed CVD 103-HgR formulation in immunocompromised populations. Persons with altered immunocompetence and their clinicians should consider the risks associated with traveling to areas with active cholera transmission. Consultation with a specialist in immunology or infectious diseases should be considered if travel to an area with active cholera transmission is necessary. ACIP generally advises against administering live vaccines to persons with most forms of altered immunocompetence (https://www.cdc.gov/vaccines/hcp/acip-recs/general-recs/immunocompetence.html).

A study of the previous formulation of CVD 103-HgR among 38 HIV-positive adults without clinical AIDS in Mali found that vibriocidal seroconversion was slightly lower among HIV-positive than HIV-negative participants (58% versus 71%) (*40*). No significant differences in occurrence of any systemic adverse events were found between vaccinated and comparison populations.

**Shedding and Transmission.** CVD 103-HgR is a live, attenuated oral vaccine that can be shed in stool for ≥7 days after vaccination and potentially transmitted to close contacts. During phase I studies of the current vaccine, the vaccine strain was cultured from stool in 11% of vaccine recipients on any day through 7 days after vaccination (*18*). In the same study, the vaccine strain was not isolated from stool collected from 24 household contacts of vaccine recipients on day 7 after vaccination (*18*). However, later transmission could have been missed.

Although handwashing after using the toilet and before preparing or handling food are part of CDC’s routine handwashing guidance (*41*), the package insert specifies that vaccine recipients should wash their hands thoroughly in these circumstances for ≥14 days after vaccination (*35*). Care providers of vaccine recipients who are diapered or who require assistance with toileting (e.g., younger children) should also follow this recommendation.

### Reporting of Vaccine Adverse Events and Additional Information

Adverse events that occur in a patient after vaccination with CVD 103-HgR should be reported to the Vaccine Adverse Event Reporting System (VAERS) regardless of whether the vaccine caused the event is certain. Instructions for reporting to VAERS can be found at https://vaers.hhs.gov/reportevent.html or by calling 1–800–822–7967. Additional information about cholera and CVD 103-HgR is available at https://www.cdc.gov/cholera/index.html.

## Future Directions

As new information becomes available on cholera epidemiology and cholera vaccines, ACIP and CDC will review and revise these recommendations as indicated. New information might include, but might not be limited to, licensure of CVD 103-HgR in other age groups or U.S. licensure of other cholera vaccines.

## References

[1] Food and Drug Administration. FDA news release: FDA approves vaccine to prevent cholera for travelers. Silver Spring, MD, US Department of Health and Human Services, Food and Drug Administration; 2016. https://www.fda.gov/news-events/press-announcements/fda-approves-vaccine-prevent-cholera-travelers

[2] Wong KK, Burdette E, Mahon BE, Mintz ED, Ryan ET, Reingold AL. Recommendations of the Advisory Committee on Immunization Practices for use of cholera vaccine. MMWR Morb Mortal Wkly Rep 2017;66:482–5. 10.15585/mmwr.mm6618a6 28493859 PMC5657988

[3] Food and Drug Administration. Supplemental approval. Silver Spring, MD: US Department of Health and Human Services, Food and Drug Administration; 2020. https://www.fda.gov/media/144754/download

[4] CDC. Cholera—*Vibrio cholerae* infection. Illness and symptoms. Atlanta, GA: US Department of Health and Human Services, CDC; 2020. https://www.cdc.gov/cholera/illness.html

[5] World Health Organization. Cholera. Geneva, Switzerland: World Health Organization; 2022. https://www.who.int/health-topics/cholera#tab=tab_1

[6] Global Task Force on Cholera Control. Cholera outbreak response field manual. Geneva, Switzerland: World Health Organization, Global Task Force on Cholera Control; 2019. https://www.gtfcc.org/wp-content/uploads/2020/05/gtfcc-cholera-outbreak-response-field-manual.pdf

[7] Ali M, Nelson AR, Lopez AL, Sack DA. Updated global burden of cholera in endemic countries. PLoS Negl Trop Dis 2015;9:e0003832. 10.1371/journal.pntd.0003832 26043000 PMC4455997

[8] Loharikar A, Newton AE, Stroika S, Cholera in the United States, 2001-2011: a reflection of patterns of global epidemiology and travel. Epidemiol Infect 2015;143:695–703. 10.1017/S0950268814001186 24865664 PMC4615619

[9] Collins J, editor. Background on cholera and CVD 103-HgR. Advisory Committee on Immunization Practices February 25, 2021 meeting. Atlanta, GA: US Department of Health and Human Services, CDC; 2022. https://www.cdc.gov/vaccines/acip/meetings/downloads/slides-2021-02/24-25/02-Cholera-Collins.pdf

[10] CDC. Travelers’ health. Cholera. Atlanta GA: US Department of Health and Human Services, CDC; 2022. https://wwwnc.cdc.gov/travel/diseases/cholera

[11] Haus-Cheymol R, Theodose R, Quilici ML, A cluster of acute diarrhea suspected to be cholera in French travelers in Haiti, December 2010. J Travel Med 2012;19:189–91. 10.1111/j.1708-8305.2012.00607.x 22530828

[12] Schilling KA, Cartwright EJ, Stamper J, Diarrheal illness among US residents providing medical services in Haiti during the cholera epidemic, 2010 to 2011. J Travel Med 2014;21:55–7. 10.1111/jtm.12075 24383654

[13] Taylor DN, Rizzo J, Meza R, Perez J, Watts D. Cholera among Americans living in Peru. Clin Infect Dis 1996;22:1108–9. 10.1093/clinids/22.6.1108 8783724

[14] Glass RI, Holmgren J, Haley CE, Predisposition for cholera of individuals with O blood group. Possible evolutionary significance. Am J Epidemiol 1985;121:791–6. 10.1093/oxfordjournals.aje.a114050 4014172

[15] Bavishi C, Dupont HL. Systematic review: the use of proton pump inhibitors and increased susceptibility to enteric infection. Aliment Pharmacol Ther 2011;34:1269–81. 10.1111/j.1365-2036.2011.04874.x 21999643

[16] Garratty G, Glynn SA, McEntire R; Retrovirus Epidemiology Donor Study. ABO and Rh(D) phenotype frequencies of different racial/ethnic groups in the United States. Transfusion 2004;44:703–6. 10.1111/j.1537-2995.2004.03338.x 15104651

[17] Tran N-T, Taylor R, Antierens A, Staderini N. Cholera in pregnancy: a systematic review and meta-analysis of fetal, neonatal, and maternal mortality. PLoS One 2015;10:e0132920. 10.1371/journal.pone.0132920 26177291 PMC4503398

[18] Chen WH, Greenberg RN, Pasetti MF, Safety and immunogenicity of single-dose live oral cholera vaccine strain CVD 103-HgR, prepared from new master and working cell banks. Clin Vaccine Immunol 2014;21:66–73. 10.1128/CVI.00601-13 24173028 PMC3910924

[19] World Health Organization. Prequalified vaccines. Geneva, Switzerland; 2022. https://extranet.who.int/pqweb/vaccines/prequalified-vaccines?field_vaccines_effective_date%5Bdate%5D=&field_vaccines_effective_date_1%5Bdate%5D=&field_vaccines_type%5B%5D=cholera%3A+inactivated+oral&field_vaccines_name=&search_api_views_fulltext=&field_vaccines_number_of_doses=

[20] Iyer AS, Harris JB. Correlates of protection for cholera. J Infect Dis 2021;224(Suppl 2):S732–7. 10.1093/infdis/jiab497 34668561 PMC8687088

[21] Ali M, Emch M, Park JK, Yunus M, Clemens J. Natural cholera infection-derived immunity in an endemic setting. J Infect Dis 2011;204:912–8. 10.1093/infdis/jir416 21849288 PMC3156915

[22] Levine MM, Black RE, Clements ML, Cisneros L, Nalin DR, Young CR. Duration of infection-derived immunity to cholera. J Infect Dis 1981;143:818–20. 10.1093/infdis/143.6.818 7252264

[23] Saha D, LaRocque RC, Khan AI, Incomplete correlation of serum vibriocidal antibody titer with protection from *Vibrio cholerae* infection in urban Bangladesh. J Infect Dis 2004;189:2318–22. 10.1086/421275 15181581

[24] Johnson RA, Uddin T, Aktar A, Comparison of immune responses to the O-specific polysaccharide and lipopolysaccharide of *Vibrio cholerae* O1 in Bangladeshi adult patients with cholera. Clin Vaccine Immunol 2012;19:1712–21. 10.1128/CVI.00321-12 22993410 PMC3491541

[25] Aktar A, Rahman MA, Afrin S, Plasma and memory B cell responses targeting O-specific polysaccharide (OSP) are associated with protection against *Vibrio cholerae* O1 infection among household contacts of cholera patients in Bangladesh. PLoS Negl Trop Dis 2018;12:e0006399. 10.1371/journal.pntd.0006399 29684006 PMC5912711

[26] Ahmed FU. US Advisory Committee on Immunization Practices (ACIP) handbook for handbook for developing evidence-based recommendations, Version 1.2. Atlanta GA: US Department of Health and Human Services, CDC; 2013. https://www.cdc.gov/vaccines/acip/recs/grade/downloads/handbook.pdf

[27] McCarty JM, Cassie D, Bedell L, Lock MD, Bennett S. Safety and immunogenicity of live oral cholera vaccine CVD 103-HgR in children aged 2–5 years in the United States. Am J Trop Med Hyg 2020;104:861–5. 10.4269/ajtmh.20-0917 33319739 PMC7941807

[28] McCarty JM, Cassie D, Bedell L, Lock MD, Bennett S. Long-term immunogenicity of live oral cholera vaccine CVD 103-HgR in adolescents aged 12–17 years in the United States. Am J Trop Med Hyg 2021;104:1758–60. 10.4269/ajtmh.20-1576 33819178 PMC8103473

[29] McCarty JM, Gierman EC, Bedell L, Lock MD, Bennett S. Safety and immunogenicity of live oral cholera vaccine CVD 103-HgR in children and adolescents aged 6–17 years. Am J Trop Med Hyg 2020;102:48–57. 10.4269/ajtmh.19-0241 31769402 PMC6947768

[30] McCarty JM, Lock MD, Bennett S, Hunt KM, Simon JK, Gurwith M. Age-related immunogenicity and reactogenicity of live oral cholera vaccine CVD 103-HgR in a randomized, controlled clinical trial. Vaccine 2019;37:1389–97. 10.1016/j.vaccine.2019.01.077 30772070

[31] McCarty JM, Lock MD, Hunt KM, Simon JK, Gurwith M. Safety and immunogenicity of single-dose live oral cholera vaccine strain CVD 103-HgR in healthy adults age 18-45. Vaccine 2018;36:833–40. 10.1016/j.vaccine.2017.12.062 29317118

[32] Chen WH, Cohen MB, Kirkpatrick BD, Single-dose live oral cholera vaccine CVD 103-HgR protects against human experimental infection with *Vibrio cholerae* O1 El Tor. Clin Infect Dis 2016;62:1329–35. 10.1093/cid/ciw145 27001804 PMC4872293

[33] Lee G, Carr W, Reingold A, ; ACIP Evidence-Based Recommendations Work Group; ACIP Evidence Based Recommendations Work Group. Updated Framework for Development of Evidence-Based Recommendations by the Advisory Committee on Immunization Practices. MMWR Morb Mortal Wkly Rep 2018;67:1271–2. 10.15585/mmwr.mm6745a4 30439877 PMC6290811

[34] Tacket CO, Cohen MB, Wasserman SS, Randomized, double-blind, placebo-controlled, multicentered trial of the efficacy of a single dose of live oral cholera vaccine CVD 103-HgR in preventing cholera following challenge with *Vibrio cholerae* O1 El tor inaba three months after vaccination. Infect Immun 1999;67:6341–5. 10.1128/IAI.67.12.6341-6345.1999 10569747 PMC97039

[35] VAXCHORA [Package insert]. Emergent BioSolutions, Gaithersburg, MD; 2022. https://www.fda.gov/media/128415/download

[36] Kollaritsch H, Furer E, Herzog C, Wiedermann G, Que JU, Cryz SJ Jr. Randomized, double-blind placebo-controlled trial to evaluate the safety and immunogenicity of combined Salmonella typhi Ty21a and *Vibrio cholerae* CVD 103-HgR live oral vaccines. Infect Immun 1996;64:1454–7. 10.1128/iai.64.4.1454-1457.1996 8606118 PMC173943

[37] Cryz SJ Jr, Levine MM, Losonsky G, Kaper JB, Althaus B. Safety and immunogenicity of a booster dose of *Vibrio cholerae* CVD 103-HgR live oral cholera vaccine in Swiss adults. Infect Immun 1992;60:3916–7. 10.1128/iai.60.9.3916-3917.1992 1500200 PMC257409

[38] McCarty J, editor. Vaxchora vaccine—pediatric dose development. Advisory Committee on Immunization Practices January 12, 2022, meeting. Atlanta, GA: US Department of Health and Human Services, CDC; 2022. https://www.cdc.gov/vaccines/acip/meetings/downloads/slides-2022-01-12/02-Cholera-McCarty-508.pdf

[39] McCarty J, editor. Vaxchora in children and adolescents. Advisory Committee on Immunization Practices February 24–25, 2021, meeting. Atlanta, GA: US Department of Health and Human Services, CDC; 2021. https://www.cdc.gov/vaccines/acip/meetings/downloads/slides-2021-02/24-25/03-Cholera-McCarty.pdf

[40] Perry RT, Plowe CV, Koumaré B, A single dose of live oral cholera vaccine CVD 103-HgR is safe and immunogenic in HIV-infected and HIV-noninfected adults in Mali. Bull World Health Organ 1998;76:63–71. 9615498 PMC2305629

[41] CDC. Handwashing in communities: clean hands save lives. When and how to wash your hands. Atlanta, GA: US Department of Health and Human Services, CDC; 2022. https://www.cdc.gov/handwashing/when-how-handwashing.html

